# Prevalence and Associated Risk Factors of *Giardia* Infection among Indigenous Communities in Rural Malaysia

**DOI:** 10.1038/srep06909

**Published:** 2014-11-04

**Authors:** Seow Huey Choy, Hesham M. Al-Mekhlafi, Mohammed A. K. Mahdy, Nabil N. Nasr, Maria Sulaiman, Yvonne A. L. Lim, Johari Surin

**Affiliations:** 1 Department of Parasitology, Faculty of Medicine, University of Malaya, 50603 Kuala Lumpur, Malaysia; 2 Department of Parasitology, Faculty of Medicine and Health Sciences, Sana'a University, Sana'a, Yemen; 3 Contagious Diseases Division, Sabah Health Department, Kota Kinabalu, Sabah, Malaysia

## Abstract

This study was carried out to investigate the prevalence and risk factors of *Giardia* infection among indigenous people in rural Malaysia. Faecal samples were collected from 1,330 participants from seven states of Malaysia and examined by wet mount and formalin-ether sedimentation methods while demographic, socioeconomic and environmental information was collected using a pre-tested questionnaire. The overall prevalence of *Giardia* infection was 11.6% and was significantly higher among those aged ≤ 12 years compared to their older counterparts. Multivariate logistic regression identified age of ≤12 years, lacking of toilet at household, not washing hands before eating, not washing hands after playing with animals, not boiling water before consumption, bathing in the river, and not wearing shoes when outside as the significant risk factors of *Giardia* infection among these communities. Based on a multilocus genotyping approach (including tpi, gdh and bg gene sequences), 69 isolates were identified as assemblage A, and 69 as assemblage B. No association between the assemblages and presence of symptoms was found. Providing proper sanitation, as well as provision of clean drinking water and proper health education regarding good personal hygiene practices will help significantly in reducing the prevalence and burden of *Giardia* infection in these communities.

G*iardia duodenalis* (syn. *G. intestinalis*; *G. lamblia*), a flagellate enteric protozoan, is the most frequently reported intestinal parasite in the world, with about 280 million people suffering from symptomatic *Giardia* infection every year^1^,^2^. It is a major cause of acute and chronic diarrhoea, particularly among children in underprivileged communities, with a prevalence range of between 10% and 50% in developing countries^3^,^4^. Moreover, it is the most common human intestinal parasite in many developed countries, with a prevalence range of between 2% and 5%^5^,^6^. The ingestion of *Giardia* cysts through contaminated food or water is the most common mode of transmission. In addition to which, person-to-person transmissions may occur through direct faecal-oral contact among family members^7^, children in day care centres^8^, and by sexual practices (oral-anal contact)^9^,^10^. The cysts are instantly infectious once they are passed out through faeces, with the potential to remain infectious for several months as they can withstand unfavourable environmental conditions^8^.

The clinical presentation of *Giardia* infection varies from an asymptomatic carrier state to a severe disease which is associated with fat malabsorption and lactose intolerance due to disaccharidase deficiency^11^,^12^. Furthermore, *Giardia* infection contributes substantially to the 2.5 million annual deaths from diarrheal disease^13^. Several studies have revealed that a chronic infection of *Giardia* during childhood contributes to protein-energy malnutrition, vitamin A deficiency, iron deficiency anaemia, zinc deficiency and poor cognitive and educational performance^14^,^15^,^16^,^17^,^18^. Socioeconomic factors such as poverty, lack of adequate sanitation and water treatment systems, illiteracy and poor hygienic practices have been identified as significant risk factors associated with *Giardia* infection in different communities^19^,^20^.

In Malaysia, data on the risk factors of *Giardia* infection is limited. However, several studies on various parasitic infections concerning the indigenous people living in Peninsular Malaysia (West Malaysia) have been enthusiastically carried out since the 1970s, these revealed that the prevalence of *Giardia* infection among Orang Asli (Aboriginal) communities could be as high as 29.2%^20^,^21^,^22^,^23^,^24^. In addition to which, *Giardia* infection has been associated with protein-energy malnutrition and vitamin A deficiency among the children in these communities^15^,^16^. Unfortunately, the majority of existing studies were conducted across a small sample size and limited geographic areas, most of which were exclusively limited to the peninsular Malaysia Orang Asli population. This makes it difficult to apply the results to a larger population, or to make strong conclusions about risk factors or control intervention. To make matters more difficult, data on *Giardia* infections in East Malaysia is not available.

It is known that *G. duodenalis* consists of eight morphologically identical but genetically distinct genotypes or assemblages, designated A–H^25^. Assemblages A and B have been identified to infect humans and many other mammalian hosts, including domestic animals and wildlife^26^, while other assemblages are host restricted; assemblages C and D infect dogs, assemblage F infects cats, assemblage E infects hoofed livestock, assemblage G infects rats, and assemblage H infects marine animals^25^,^27^. With regards to clinical manifestation, studies on the correlation between the assemblages and clinical symptoms have reported controversial results. Some studies have pointed out that symptoms are more associated with assemblage A^28^, while others have found that assemblage B infections are more likely to be symptomatic^29^. Data on the genotypes of *Giardia* in Malaysia are scarcely available^30^,^31^. Within this context, this community-based study was conducted to provide a comprehensive dataset regarding the prevalence and potential factors associated with *Giardia* infection, as well as the genotypes of *Giardia* among different indigenous groups in Malaysia, with a preference towards evaluating the connection of *Giardia* infection in indigenous groups with socioeconomic status in rural Malaysia.

## Results

### Study cohort and socioeconomic profile

A total of 1330 participants (50% males, 50% females) with a mean age of 15 years from seven states of Malaysia (986 from Peninsular Malaysia and 344 from Sabah, East Malaysia) were enrolled in the study. Among the cohort from Sabah, 175 (50.9%) were from the Dusun tribe, 97 (28.2%) from the Murut tribe, and 72 (20.9%) from the Bajau tribe. Among the cohort from Peninsular Malaysia, 484 (49.1%) were from the Semai tribe, 268 (27.2%) from the Temuan tribe, 99 (10.0%) from the Temiar tribe, 68 (6.9%) from the Jahut tribe, and 67 (6.8%) from the Kensiu tribe. Overall poverty prevails in these communities, with almost half (50.9%) of all families having a low monthly income (under RM500, the poverty income threshold in Malaysia). The greatest number of families with household incomes of less than RM500 is located in Peninsular Malaysia (56.6%), with Sabah also struggling with a spread low income households (34.0%).

With regard to educational status, 62.9% of the participants had at least primary levels of education, with a higher percentage of subjects in Sabah being educated when compared to Peninsular Malaysia (75.7% vs 58.5%). Almost half of the adult participants are not working (48.6%), with almost similar frequencies of unemployment in both Peninsular Malaysia and Sabah (49.7% vs 46.8%). Those working were mainly farmers (39.5%) or otherwise engaged in agriculture (25.6%) (rubber and oil palm plantations), forestry, fishing and related occupations. General characteristics of the participants are presented in Table 1.

### Prevalence of *Giardia* infection

The overall prevalence rate of *Giardia* infection was 11.6% (154/1330), with a significantly higher infection rate in Peninsular Malaysia when compared to Sabah (13.6%; 95% CI = 11.6, 15.9 vs 5.8%; 95% CI = 3.8, 8.8; *P* < 0.001). The prevalence of infection was also significantly higher among participants aged under 12 years of age (14.2%; 95% CI = 12.1, 16.6 vs 5.5%; 95% CI = 3.7, 8.2; *P* < 0.001). There was a similar prevalence of infections reported among both males and females (12.1%; 95% CI = 9.8, 14.8 vs 11.1%; 95% CI = 8.9, 13.8; *P* = 0.593). With regards to the tribes in Peninsular Malaysia, the prevalence of *Giardia* infection was significantly higher among participants from the Semai tribe (17.8%), followed by the Kensiu (13.4%) and Temuan (10.8%) tribes. Similarly, the amount of infections were higher among the Dusun tribe of Sabah (8.6%) compared to the Murut and Bajau tribes. At the level of states, the highest prevalence of infections were reported in Pahang (15.9%) followed by Negeri Sembilan (14.9%) and Kedah (13.4%), while the lowest reported levels of infections were in Malacca (4.6%).

Faecal specimens were also screened for the presence of other intestinal parasitic infections, with participants being found to be infected with *Trichuris trichiura* (54.0%), *Ascaris lumbricoides* (28.7%), *Entamoeba histolytica/dispar/moshkovskii* (16.5%), and hookworm (10.5%). Overall, the prevalence of all detected infections was significantly higher among the participants from Peninsular Malaysia compared to those from Sabah (*P* < 0.001). About two-thirds of *Giardia* cases (104/154) were mixed infections with one or more parasite species, while one third were *Giardia* single infection (50/154). Regarding co-infections, *Giardia* and *Trichuris* was the most common co-infection, followed by *Giardia* with *Ascaris* and *Giardia* with *Entamoeba* species. The prevalence and distribution of infections according to location and tribes are shown in Table 2.

### Associated factors with *Giardia* infection

The associations of *Giardia* infection with demographic, socioeconomic and environmental factors are illustrated in Table 3. Besides location (Peninsular Malaysia) and age (≤12 years), participants from large households (with family sizes numbering more than 7 members living together) experienced a significantly higher prevalence of *Giardia* infection than those from smaller families (14.8% 95% CI = 12.1, 18.5 vs 10.0%; 95% CI = 8.2, 12.0).

There was also a significant association between *Giardia* infection and subjects educational level, with a higher prevalence among those who were either non educated or only had primary education when compared to those who had a secondary education (12.3% 95% CI = 10.6, 14.3 vs 4.7%; 95% CI = 2.2, 9.8). However, prevalence of *Giardia* was not significantly different between those who had a primary education (12.7%; 95% CI = 10.5, 15.4) and those who were non educated at all (11.8%; 95% CI = 9.8, 15.0).

Furthermore, the prevalence of *Giardia* infection was significantly higher among those who live in houses without toilets when compared to those living in houses with functioning toilets (17.4%; 95% CI = 13.7, 21.9 vs 9.7%; 95% CI = 8.0, 11.7). With regards to hygienic practices, the results of univariate analyses revealed that the prevalence of *Giardia* infection is significantly associated with not washing hands before eating, not boiling water before consumption, bathing in the river, indiscriminate defecation, not washing vegetables/fruits before consumption, not wearing shoes when outside, not washing hands after playing with animals and indiscriminate garbage disposal.

Interestingly, when we stratified the univariate analyses according to location, a significantly higher prevalence of *Giardia* infection was found among those drinking pipe water when compared to those who collect drinking water from unsafe sources (15.9% vs 11.5%; χ^2^ = 4.181; *P* = 0.041). It was found that participants from East Malaysia who still collect their drinking water from rivers, wells and rain have a higher prevalence of infection when compared to their counterparts; however the difference was not statistically significant (15.8% vs 5.2%; χ^2^ = 3.655; *P* = 0.056).

Overall, all the significant associations were retained by the Peninsular Malaysia group, while only not washing vegetables/fruits before consumption (OR = 3.4; 95% CI = 1.1, 11.1) was retained as a significant variable in regards to the Sabah group.

### Risk factors of *Giardia* infection

Table 4 shows that a multivariable logistic regression model retained 7 variables as being significant risk factors in terms of inducing *Giardia* infection among the studied indigenous people. The results confirmed that those aged ≤ 12 years (OR = 2.1) and living in a house without a functioning toilet (OR = 1.5) were at higher odds of having a *Giardia* infection when compared with their counterparts. Moreover, poor personal hygiene practices, including not washing hands after playing with animals, not boiling water before consumption, bathing in the river, not wearing shoes when outside and not washing hands before eating were also retained as significant risk factors of *Giardia* infection among these people.

### Association of *Giardia* infection with symptoms

The majority of participants, 1252 (94.1%), had no complaints about gastrointestinal signs or symptoms. Of 78 symptomatic cases, 34.6% (27/78) had diarrhea, 46.2% (36/78) had diarrhea and abdominal pain, 6.4% (5/78) had abdominal pain, 10.3% (8/78) had vomiting, and 2.6% (2/78) had dysentery. Most *Giardia*-positive cases in this study were asymptomatic (129 out of 154 cases), and about one-third (25/78) of the symptomatic individuals were infected with *Giardia*. The prevalence of infection was significantly higher among the 63 participants who had diarrhea when compared to their asymptomatic counterparts (36.5% vs 10.3%; χ^2^ = 40.142; *P* < 0.001). Similarly, the prevalence of infection was higher among those who had abdominal pain compared to the asymptomatic individuals, however the difference was not statistically significant (19.5% vs 11.3%; χ^2^ = 2.601; *P* = 0.107). It was noted that 57 (90.5%) of the diarrhea cases were from Peninsular Malaysia, compared to only 6 (9.5%) cases from Sabah (χ^2^ = 9.209; *P* = 0.002).

### Molecular characterization of *G. duodenalis*

Analysis using multilocus genotyping approach on the positive isolates had successfully amplified 138 samples using at least one of the following markers: triose phosphate isomerase (tpi), glutamate dehydrogenase (gdh), and beta-giardin (bg) genes. When sequences generated from the amplicons were analyzed using multiple alignment with previously published reference sequences, 69/138 (50.0%) were genotyped as assemblage A and 69/138 (50.0%) were genotyped as assemblage B. The distribution of both assemblages was almost similar between the West and East Malaysia (*P* = 0.613).

Gastrointestinal symptoms were reported in 18.8% (13/69) of the individuals infected with assemblage A; 12 of the cases had diarrhea. Likewise, 14.5% (10/69) of the individuals infected with assemblage B were symptomatic and had diarrhea. That said, among *Giardia*-positive and symptomatic individuals (i.e. 23 individuals), the prevalence of assemblages A and B was 56.5% and 43.5% respectively, and the difference was not significant (OR = 1.37; 95% CI = 0.56, 3.38).

## Discussion

Despite intensive efforts to improve the quality of life in rural Malaysian communities, intestinal parasitic infections including giardiasis, amoebiasis and soil-transmitted helminthiasis are still highly prevalent, especially among aboriginal and rural populations. The present study provides information on the status of *Giardia* infection among different indigenous people in rural Malaysia, including the Orang Asli (aboriginal) population in West Malaysia and other indigenous groups in East Malaysia. Our findings revealed that the overall prevalence of *Giardia* infection was 11.6%, with a significantly higher prevalence among the participants from West Malaysia when compared to those from East Malaysia (13.6% vs 5.8%). This is in agreement with a previous study among 716 rural individuals from five states of West Malaysia^32^. Furthermore, several small-scale studies have been conducted among the aboriginal population in West Malaysia which showed that the prevalence of *Giardia* ranges between 4.0% and 29.2%^24^,^30^,^31^,^32^,^33^,^34^. In addition to which, a recent study among three aboriginal tribes in three different states of West Malaysia revealed a high prevalence (20.0%) of *Giardia* infections^20^.

In West Malaysia, our findings showed that the prevalence of *Giardia* infection was highest among participants from Pahang state (15.9%) followed by Negeri Sembilan (14.9%) and Kedah (13.4%), while the lowest levels of prevalence were found among the participants from Malacca 4.6% and Selangor 6.1%. This could be attributed to the differences in the culture, environment and population of Orang Asli communities in these areas. Orang Asli villages in Pahang, Kedah and Negeri Sembilan are located deep in the jungle, with inadequate sanitary facilities and away from healthcare facilities. In contrast, Selangor and Malacca are in peripheral areas with better sanitation and environmental factors, as well as being located near to the region's main health facilities. Our findings further revealed that the Senoi group (Semai, Jahut and Temiar tribes) had the lowest prevalence of infection amongst its population (9.6%), while the prevalence among the Negrito group (Kensiu) was higher than the Proto-Malay group (Temuan) (13.4% vs 10.8%).

These findings are consistent with previous studies conducted among the different ethnic groups of Orang Asli in West Malaysia, which reported a higher prevalence of *Entamoba* species and STH among Negrito groups, followed by Senoi and Proto-Malay groups^35^,^36^. Similarly, an earlier study among 1,273 individuals from different ethnic groups showed that the Negritos harboured more intestinal parasites species when compared with other ethnic tribes^37^. Orang Asli belonging to the Negrito group live in remote areas, with their villages being made of wood or bamboo, which means they suffer from poor housing conditions, a lack of proper sanitation and no provisions for a clean water supply when compared to the Senoi or Proto-Malay groups who live in suburban areas under better conditions.

With regards to East Malaysia (Sabah and Sarawak states), supporting information on the prevalence of intestinal parasitic infections is lacking. Our findings showed that the prevalence of *Giardia* infection in Sabah was much lower (5.8%) than in West Malaysia, with a significantly higher prevalence among participants from the Dusun tribe compared to those from Murut and Bajau tribes. Unfortunately, the only previous report from East Malaysia was conducted solely in Sarawak, revealing a very low prevalence of *Giardia* infection (2.0%) among 355 individuals from five interior communities^38^. The government has made intensive efforts to improve the quality of life of indigenous people throughout the country, with their main strategy being to the reallocate those living in remote areas to new settlements at the periphery of towns. New houses were provided to the settlers which ensured access to basic amenities, which has helped greatly reduce the prevalence of many parasitic infections amongst the indigenous communities in Sabah. On the other hand, the adherence of the Orang Asli people in West Malaysia to their jungle habitats has constrained the overall effectiveness of this strategy. Therefore, providing new houses within the same locality will not help reduce the prevalence of intestinal parasitic infections in this area due to the heavily contaminated environment.

The present study investigated the possible risk factors associated with *Giardia* infection among the participants, revealing that children under 13 years old (1 month to 12 years old) were significantly associated with higher *Giardia* infection rates when compared to adults and children over 12 years old (13 to 84 years old), and this is in agreement with previous studies^22^,^24^,^31^. This could be attributed to the higher exposure of young children to the source of a wide range of infections, which could be due to having lower standards of personal hygiene when compared to the adults and older children.

In addition, the research revealed that not boiling drinking water before consumption was reported as a significant predictor of *Giardia* infection. It is well documented that *Giardia* and *Cryptosporidium* have been the most common causes of waterborne diseases outbreaks worldwide^39^. Our findings further showed that living in houses without functioning toilets increased the odds of the observed population acquiring *Giardia* infections, and this is consistent with many previous studies in Malaysia and elsewhere^32^,^40^,^41^. It was further found that defecating in indiscriminate places, such as in rivers and bushes was a common practice in communities with inadequate toilet facilities.

In general, Orang Asli have a habit of building their villages beside rivers where water can be conveniently collected for multiple purposes, including drinking and cooking, whilst also conducting daily activities such as bathing and washing clothes in the river water. Besides which some inhabitants, especially children, prefer defecating at the site of the stream^40^. As a result, it is likely that the environment of the local rivers are heavily contaminated, becoming a source of infection for *Giardia* and other intestinal parasites/bacteria/viruses^23^,^40^. This presumption is supported by a finding from the present study, in which bathing in the rivers appeared as one of the significant risk factors. In the same vein, an interesting finding was uncovered when we stratified the univariate analyses according to location, in which a significantly higher prevalence of *Giardia* infection was found among Orang Asli who drink piped water when compared to those collected drinking water from unsafe sources (i.e. rivers, wells and rain) (15.9% vs 11.5%; χ^2^ = 4.181; *P* = 0.041). In contrast, participants from East Malaysia who use piped water for drinking had a lower prevalence of infections when compared to their counterparts who used unsafe water (5.2% vs 15.8%; χ^2^ = 3.655; *P* = 0.056). Drinking piped water has been identified as a significant risk factor among the aboriginal population in Pahang, Malaysia^32^. However, results from previous studies conducted in these communities showed that treated water is free from faecal coliforms, *Giardia* and *Cryptosporidium* contamination^42^,^43^. Hence, we believe that the contamination of the piped water is occurring after the treatment process has taken place, and could be attributed to the usage of containers and utensils that may have been previously soiled with *Giardia* cysts when handling and storing drinking water. Furthermore, it was observed that the Orang Asli people in West Malaysia who used tanks or other containers for drinking water collection left these containers uncovered, which is in direct contrast to the clean and covered tanks in East Malaysia.

Our findings also showed that not washing hands before eating or after handling/playing with animals were significant risk factors for *Giardia* infection among the study population. Although previous studies have suggested zoonotic transmission for *Giardia* infection, studies among Malaysian aborigines have previously found no such association^44^,^45^. *Giardia* cysts can remain infective in the environment for a very long period of time, meaning they could easily get picked-up on the fur of animals such as cats and dogs, who were observed as moving about in the contaminated environment while also mixing freely with the members of their households. Hence, not washing hands after handling or playing with these animals could facilitate the spread of the infections. Similarly, not wearing shoes when outside the house may also contribute to the contamination of houses with cysts, and this was also identified as a significant predictor of *Giardia* infection by the present study.

It is also worth noting that a significantly higher prevalence of *Giardia* infection was reported among participants with low educational levels (either non educated or with only primary levels of education), as well as in areas in which indiscriminate defecation was common, in areas with indiscriminate garbage disposal systems, in large households (>7 members), and when not washing vegetables/fruits before consumption. However, these associations were not retained by the logistic regression model. Previous studies among aboriginal communities in West Malaysia have identified these variables as significant predictors of *Giardia* and other intestinal parasitic infections^20^,^31^,^32^,^46^.

In view of this, when analysing the risk factors according to location, it is remarkable to find out that all of the variables were retained as significant risk factors of *Giardia* infection in West Malaysia, while only one significant risk factor was retained among the participants from East Malaysia (that is not washing vegetables/fruits before consumption). It is also interesting to note that while eating raw vegetables and fruits has been identified as significant risk factors of *Giardia* infection among Orang Asli in West Malaysia^31^,^47^, our study revealed that the significant association is actually in the practice of not washing vegetables or fruits prior to consumption, which appeared to be a more tenable reason when compared to the simple consumption of this nutritious food. Most of the fruits in these communities are tropical peeled fruits like rambutan (*Nephelium lappaceum*), longan (*Dimocarpus longan*) and mangosteen (*Garcinia mangostana*). We observed people collecting dropped fruits (rambutan, longan) from the ground, opening the soft shell with their mouths and eating the fruit directly without first washing it. Thus, fruits and vegetables could be the medium of transmission in cases where the surface carries the parasites infective stages, especially when the fruit has been in contact with the contaminated ground followed by the direct consumption of the fruit itself, both of which increase the chances of transmission from contaminated hands.

The molecular findings of the current study showed that assemblages A and B were present at equal frequency (69/138). Indeed, our findings contradict previous genotyping data using a single locus (i.e. *tpi* gene)^30^,^31^. A recent community-based study identified two-thirds of 98 *Giardia*-positive isolates as assemblage A and the rest were assemblage B^30^. By contrast, a previous study using SSU rRNA locus identified only one specimen as assemblage A in 42 specimens while the rest were assemblage B^31^. This is, however, different from the proportion of assemblages A and B reported globally where assemblage B (~58%) has a higher prevalence than assemblage A (~37%)^48^.

We found a significant association between *Giardia* infection and diarrhoea among the studied population, with significantly higher frequency of diarrhoeal cases among *Giardia*-infected participants from West Malaysia when compared to their counterparts from East Malaysia. However, about two-thirds of the *Giardia* cases were mixed infection with at least one parasite species. Therefore, it was not possible to confirm the causal relationship between *Giardia* and diarrhoea in the present study, due to the limitation of the cross-sectional design used to gather results. With regard to genotypes, gastrointestinal symptoms such as diarrhea, vomiting, abdominal pain and dysentery were reported in 18.8% and 14.5% of assemblage A and B respectively. We found no significant difference in the prevalence of both assemblages among symptomatic infections. Similar findings were also reported by previous studies^49^,^50^,^51^,^52^. By contrast, a previous study among Orang Asli in Malaysia reported a strong association between the clinical symptoms of gastroenteritis and assemblage B^47^. To date, there is still a lack of clear association between the assemblage and the clinical outcome, with contradictory results. While previous studies conducted in Bangladesh, Australia and Spain have reported a significant association between assemblage A and the presence of symptoms^28^,^53^,^54^, other studies from various regions also suggest a correlation between the presence of symptoms and infection with assemblage B^29^,^55^,^56^,^57^,^58^. Hence, conclusive inference with regard to the genotype-dependent pathogenecity may only be drawn after further well-designed studies^12^.

Besides that, the present study showed that the majority of *Giardia*–positive individuals were asymptomatic (83.8%). This high percentage of asymptomatic infections should be taken with apprehension from the public health standpoint as the infected individuals can act as carriers and excrete infective cysts in faeces. Infection is acquired by ingestion of the resilient cysts via contaminated food, water or hands. It could have an adverse impact especially to the family and the community, if the symptom-free cases go unnoticed and contaminate the environment. The cysts have been reported to have a low infectious dose of as few as ten cysts to establish an infection and become immediately infectious upon being released in stools^59^. More significantly, this health hazard could be long lasting because the transmissive stage of this parasite can persist between 7 to 18 days in faeces, 7 weeks in soil and up to 3 months in water^60^,^61^. Yet there has been no study conducted to investigate what genotype of *Giardia* infection sheds higher number of cysts or has higher level of resistance to withstand the external environment and maintain longer periods of infectivity. If these factors are genotype-related, one will expect that a particular assemblage will exist in greater amounts in the environment and potentially lead to a higher chance of transmission. One relevant study by Haque et al. reported that assemblage B infections produced a higher load of DNA and higher overall prevalence^28^.

The present study provides a community-based picture on the epidemiology of *Giardia* infection among the indigenous people in rural Malaysia. Overall, our findings show that *Giardia* infection in these communities was mostly associated with poor hygienic practices that were often coupled with poor sanitary facilities as well. From the general characteristics of both groups, it is clear that the indigenous people in East Malaysia are more educated, have a higher monthly household income, live in better housing conditions and have a cleaner environment when compared to the indigenous people in West Malaysia. Hence, the lifestyle interventions already implemented in East Malaysia might be the explanation as to why there is a significantly lower prevalence of *Giardia* reported in that region.

We do acknowledge some limitations of the present study. In many cases only a single faecal sample was collected, instead of the ideal three consecutive samples, because of a limitation of resources and the cultural belief of some Orang Asli against giving their faecal samples. Therefore, the prevalence of *Giardia* infection might be underestimated due to the variation in cyst shedding per days. Many indigenous villages are located in deeply remote areas, with no road access, and therefore were not involved in our study, though it is worth noting that even higher prevalence rates of intestinal parasitic infections have been previously reported in these remote areas when compared to the villages involved in our study^33^. We speculate that our findings can be generalised to other rural indigenous children in other states. That said, it is possible that these findings may not be generally applicable to the entire Malaysian rural population, as ethnic groups other than the indigenous people tend to have better socioeconomic and environmental situations. Hence, further studies are required in order to confirm these conjectures.

In conclusion, the findings reveal that the prevalence of *Giardia* infection is still high and of public health concern among indigenous populations in rural Malaysia. The prevalence was found to be higher among the aboriginal population in West Malaysia when compared to the indigenous people in East Malaysia. It was most commonly found among children and those living in poor sanitation conditions who also had poor standards of personal hygiene. Different control measures are required in order to combat current levels of infection, including health education pertinent to good personal hygiene and good sanitary practices, as well as education aimed at improving general awareness about parasitic infections. Besides which, if re-allocation to new settlements is not possible, providing proper sanitation, as well as making provisions for clean and safe drinking water, are crucial for maintaining the health of indigenous communities in West Malaysia.

## Methods

### Study areas

**Figure 1:**
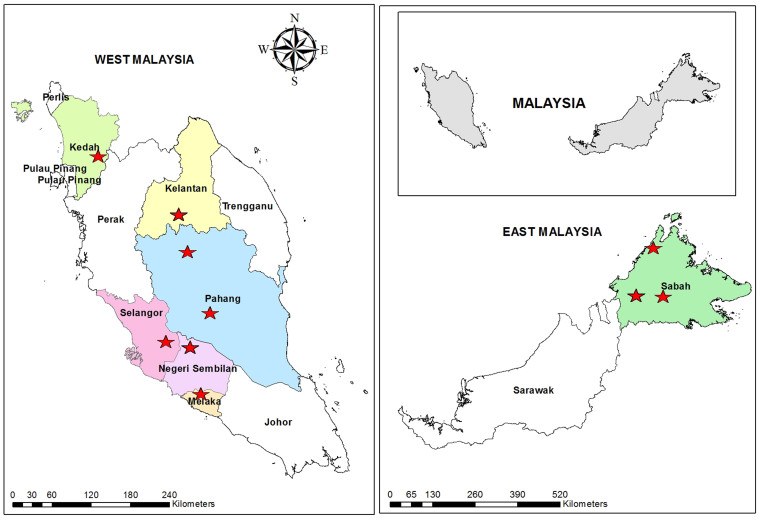
A geographic map showing the location of the districts (stars) and states (coloured) involved in the study. The map was created using the Esri ArcMap 10.2.1 software.

This community-based cross-sectional study was carried out among indigenous communities in rural parts of Malaysia (both in West and East Malaysia) from April 2011 to February 2013. Overall, 28 villages from seven states of Malaysia, namely Pahang, Selangor, Negeri Sembilan, Kelantan, Kedah, Malacca and Sabah were involved (Figure 1). The populations in the areas under study lived with disparate lifestyles and environmental exposures, especially in terms of the indigenous groups in Peninsular Malaysia (West Malaysia) and Sabah (East Malaysia). In particular, the overall living standard was lower among the indigenous groups in West Malaysia compared to their counterparts in East Malaysia, though the housing conditions vary among the villages. The climate is equatorial with hot-humid conditions and rainfall throughout the year. The vegetation is the thick rain forest type and there are few water streams in the area.

### Study population

The aboriginal communities in West Malaysia are recognized specifically as ‘Orang Asli', a collective Malay term translated as ‘original or first people'. This term is used to address the heterogeneous minorities that are classified in to three main groups, namely Negrito (2.8%), Proto-Malay (42.3%) and Senoi (54.9%). Each of these groups consists of six tribes, with an estimated population of 178,197, which makes up 0.7% of the total Malaysian population according to the population census 2010. In contrast with West Malaysia, the indigenous peoples form more than 50.0% of the total population of Sabah and Sarawak (East Malaysia). In Sabah, there are 72 ethnic and sub-ethnic groups, with Kadazandusun (17.7%), Bajau (14.0%) and Murut (3.2%) making up the major composition of the Sabah population^62^.

The lifestyles and means of pursuing a livelihood are diverse among the indigenous groups, though they tend to have a close connection with the various tribes traditional habitats and natural resources. Some of the aborigines in the Peninsular Malaysia still live in remote areas, however, due to implementation of development programmes that were initiated by the government, an increasing number of these minorities are moving to the periphery of urban areas where they are integrating with urban communities. The common economic activities engaged in by the present Orang Asli are rubber tapping, small-scale cultivation of local crops (e.g. cassava and banana), wage-earning jobs in private sectors (e.g. oil palm/coco plantation, construction site and factory), as well as forest produce collection and selling (e.g. fruits and bamboo) to a lesser extent^63^. Conversely, the indigenous communities in the rural areas of Sabah employ a more diversified subsistence economy. The coastal and riverine communities engage largely in the fishing industry, with a recent expedition in to seaweed cultivation. Whilst those living in the interior areas depend on farming, gardening and collecting forest resources, both for their own consumption and in order to sell the surplus for cash. Some of them are involved in cash crops plantation projects, such as oil palm, cocoa and rubber.

### Sample size and sampling strategy

The minimum sample size required for this study was calculated according to the formula provided by Lwanga and Lemeshow^64^. At a 5% level of significance and a 95% confidence level, the minimum number of subjects required for this study was estimated as being 983, assuming that the prevalence of *Giardia* infection was 20%; as recently reported among three different Aboriginal tribes^20^. Overall, 1,330 individuals agreed to voluntarily participate in this study who met the inclusion criteria (signed written consent, completed questionnaire and delivered stool samples for examination).

The states and villages involved in this study were randomly selected from the available official administrative list in collaboration with the Department of Orang Asli Development (Jabatan Kemajuan Orang Asli, JAKOA) and Sabah Health Department (JKN SABAH), with consideration of the following criteria for villages: located in a rural area, accessible from the main roads and each village has more than 20 houses or ≥100 residents. The probability proportional to size sampling method was used to select the participants from each state, based on the total number of the indigenous population in the states and districts. Before the commencement of the sampling surveys, the villages were visited by researchers accompanied by officers from JAKOA and JKN SABAH, to meet the head of the village so as to explain and prepare the villagers for data and sample collections, as well as to obtain primary information on the existing conditions in the villages.

### Questionnaire survey

A pretested questionnaire was used to collect information on the demographic (e.g. age, sex and number of household members), socioeconomic (e.g. household monthly income, occupation and educational status), environmental (e.g. availability and types of toilets in the household, types of water supply, garbage disposal and presence of domestic animals), personal hygiene (e.g. washing hands before eating, after defecation and after playing with animals, washing vegetables and fruits before consumption, boiling water before consumption and bathing place), and general health status of the participants(i.e. symptoms related to intestinal parasitic infections such as diarrhoea, nausea, vomiting, abdominal pain and a history of receiving anthelmintics treatment). The questionnaire was designed in the English language and then translated into the Malay language. Two research assistants from the Department of Parasitology, University of Malaya were trained for the purpose of this study.

### Faecal samples collection and examination

Fresh faecal samples were collected in 60 ml clearly labelled containers with wide mouths and screw-caps. The containers were distributed and the participants were informed on the proper method of sample collection e.g. not to mix their stool sample with urine or water, and to provide a sample amount of at least a thumb-size. Participants were invited to bring their early morning stool samples the next day. Samples were transported in suitable cool boxes to the Department of Parasitology, Faculty of Medicine, University of Malaya and stored in a cold room at 4°C. When immediate transfer of samples to the department was not possible, samples were kept in a refrigerator at local clinics or health offices.

The collected faecal samples were processed based on the formalin-ether sedimentation technique previously described by Cheesbrough^65^. Pellets of the sediment were used for examination by emulsifying them in 1–2 drops of iodine solution, then examining the results using a light microscope with the aim of finding the presence of *Giardia* cysts and/or trophozoites, as well as other intestinal parasites. For quality control, duplicate slides were prepared from 20% of the samples and the slides were read by another microscopist.

### Molecular analysis

DNA extraction and molecular analysis were carried out using PCR relevant protocols published elsewhere^66^. In brief, DNA was extracted directly from faecal samples either using PowerSoil®DNA Isolation Kit (MoBio Laboratories Inc., Carlsbad, California) or MACHEREY-NAGEL NucleoSpin® Soil (MACHEREY-NAGEL GmbH & Co. KG, Düren, Germany) to a final volume of 50 μl. Multilocus genotyping was performed using markers that amplify 432-bp fragment of the *gdh* gene^54^, 530-bp fragment of the *tpi* gene^67^ and 511-bp fragment of the *bg* gene^68^,^69^ to analyse microscopy *Giardia*- positive samples.

### Statistical analysis

Data analysis was done by using SPSS for WINDOWS (version 18.0). Data was entered and reviewed by two different researchers. For descriptive analysis, the prevalence of infections and other categorical variables were expressed in percentages, while mean (standard deviation; SD) was used to present the quantitative data, with results being presented in tables. Pearson's Chi Square test was used to investigate the association between *Giardia* infection as the dependent variable and demographic, socioeconomic, environmental and personal hygiene factors as the independent variables. All the variables used in the survey were coded in a binary manner as dummy variables. For example, *Giardia* infection (positive = 1, negative = 0); gender (male = 1, female = 0); availability of piped water supply of toilet in the house (no = 1, yes = 0) and washing hands before eating (no = 1, yes = 0). Family size was categorized into two groups (>7 and ≤7 members), and age of participants was also categorized into two groups (≤12 and >12 years)^23^,^31^,^32^. Furthermore, a monthly household income of <RM500 was considered as being low based on the poverty income threshold in Malaysia^32^. Odd ratios (OR) and 95% confidence intervals (CI) were calculated using univariate and multivariable logistic regression analyses. All tests were considered significant at *P* < 0.05.

### Ethical consideration

The protocol of this study was approved by the Medical Ethics Committee of the University of Malaya Medical Centre (Ref. no: 788.74 and 878.19). Prior to the commencement of the survey questionnaire and sample collection, permissions were obtained from the heads of the villages. Then, the participants were informed about the objectives and methods of the study. They were informed that their participation was totally voluntary and that they could withdraw from the study at any time without citing any reason whatsoever. Written and signed or thumb-printed informed consent was obtained from those who agreed to participate, or from parents or guardians on behalf of their children, and these procedures were approved by the Medical Ethics Committee of the University of Malaya Medical Centre. The methods used in this research were carried out in accordance with the approved guidelines.

## Author Contributions

J.S., H.M.A., M.A.K.M. and S.H.C. conceived and designed the experiments. S.H.C. and N.A.N. collected the samples and performed the experiments. S.H.C. and H.M.A. analyzed the data and wrote the paper. M.S. provided logistic support for data collection and field work. H.M.A., J.S. and Y.A.L.L. revised the article critically for important intellectual content.

## Figures and Tables

**Table 1:** General characteristics of the indigenous communities that participated in this study

Characteristics	Peninsular Malaysia (n = 986)	Sabah (n = 344)	Overall (n = 1330)
Age group			
≤12 years (1 month–12 years)	723 (73.3)	207 (60.2)	930 (69.9)
>12 years (13–84 years)	263 (26.7)	137 (39.8)	400 (30.1)
Gender			
Male	500 (50.7)	164 (47.7)	664 (49.9)
Female	486 (49.3)	180 (52.3)	666 (50.1)
Socioeconomic status			
Low household income (<RM500)	558 (56.6)	119 (34.6)	677 (50.9)
>7 members-large	353 (35.8)	79 (23.0)	432 (32.5)
Not working	100 (50.5)	60 (47.6)	160 (49.4)
Educational level			
Secondary education	66 (6.7)	63 (18.3)	129 (9.7)
Primary education	512 (51.9)	196 (57.0)	708 (53.2)
Non educated	408 (41.4)	85 (24.7)	493 (37.1)
Supplied with piped water	471 (47.8)	325 (94.5)	796 (59.8)
Presence of toilet at household	706 (71.6)	296 (86.0)	1002 (75.3)
Presence of animals at household	698 (70.8)	245 (71.8)	943 (71.1)

**Table 2:** Prevalence and distribution of intestinal parasitic infections among the indigenous communities that participated in this study

Parasite	*Giardia*	*Trichuris*	*Ascaris*	*Entamoeba*	Hookworm
States					
Selangor	6.1	83.7	57.1	18.4	0.0
Malacca	4.6	53.8	27.7	9.2	0.0
Negeri Sembilan	14.9	61.7	34.4	5.2	11.0
Kedah	13.4	62.7	61.2	32.8	14.9
Pahang	15.9	69.9	40.6	27.0	17.6
Kelantan	8.1	9.1	3.0	15.2	1.0
Sabah	5.8	1.2	4.4	2.9	4.1
Tribes					
Semai	17.8	71.7	37.4	29.8	17.6
Kensiu	13.4	62.7	61.2	32.8	14.9
Jahut	2.9	57.4	63.2	7.4	17.6
Temiar	8.1	9.1	3.0	15.2	1.0
Temuan	10.8	63.8	36.9	8.6	6.3
Dusun	8.6	0.0	0.0	2.9	4.0
Murut	3.1	4.1	15.5	3.1	7.2
Bajau	2.8	0.0	0.0	2.8	0.0
Overall prevalence	11.6	46.0	28.7	16.5	10.5

**Table 3:** Univariate analysis of factors associated with *Giardia* infection among the indigenous communities that participated in this study

Variables	No. Examined	No. Infected	% Infected	OR	95% CI	*P*-value
Location						
Peninsular Malaysia	986	134	13.6	2.5	1.6, 4.1	<0.001
Sabah (East Malaysia)	344	20	5.8	1		
Age group						
< = 12 Years	930	132	14.2	2.8	1.8, 4.5	<0.001
>12 Years	400	22	5.5	1		
Gender						
Male	664	80	12.0	1.1	0.8, 1.5	0.593
Female	666	74	11.1	1		
Size of household						
>7 members	432	64	14.8	1.6	1.1, 2.2	0.011
< = 7 members	898	90	10.0	1		
Household income						
<RM500	677	88	13.0	1.3	0.9, 1.9	0.099
> = RM500	653	66	10.1	1		
Educational level						
Secondary education	129	6	4.7	1		
Primary education/non educated	1201	148	12.3	2.9	1.2–6.7	0.010
Employment status						
Not working	160	5	3.1	0.6	0.2, 2.0	0.421
Working	164	8	4.9	1		
Source of drinking water						
Unsafe water (river, well, rain)	534	62	11.6	1.0	0.7, 1.4	0.977
Piped water	796	92	11.6	1		
Presence of toilet at household						
No	328	57	17.4	2.0	1.4, 2.8	<0.001
Yes	1002	97	9.7	1		
Boiling water before consumption						
No	192	40	20.8	2.4	1.6, 3.5	<0.001
Yes	1138	114	10.0	1		
Bathing place						
River	279	52	18.6	2.1	1.5, 3.1	<0.001
Bathroom	1049	102	9.7	1		
Indiscriminate defecation						
Yes	437	77	17.6	2.3	1.6, 3.2	<0.001
No	890	77	8.7	1		
Washing hands before eating						
No	327	49	15.0	1.5	1.0, 2.2	0.028
Yes	1003	105	10.5	1		
Washing hands after defecation						
No	269	35	13.0	1.2	0.8, 1.8	0.420
Yes	1058	119	11.2	1		
Consumption of raw vegetables						
Yes	453	45	9.9	0.8	0.5, 1.1	0.171
No	874	109	12.5	1		
Washing vegetables/fruits before consumption						
No	335	59	17.6	1.9	1.3, 2.9	<0.001
Yes	993	95	9.6	1		
Wearing shoes when outside						
No	316	58	18.4	2.2	1.5, 3.1	<0.001
Yes	1014	96	9.5	1		
Garbage disposal						
Indiscriminate	452	75	16.6	2.0	1.4, 2.8	<0.001
Proper disposal	875	79	9.0	1		
Presence of domestic animals						
Yes	943	117	12.4	1.3	0.9, 2.0	0.153
No	384	37	9.6	1		
Washing hands after playing with animals						
No	266	50	18.8	2.1	1.5, 3.1	<0.001
Yes	1061	104	9.8	1		

**Table 4:** Multivariate analysis of risk factors associated with *Giardia* infection among the indigenous communities that participated in this study

Variables	Adjusted OR	95% CI	*P*-value
Location (Peninsular Malaysia)	1.5	0.9, 2.6	0.115
Being aged ≤ 12 years	2.1	1.3, 3.4	0.003
Gender (males)	1.1	0.7, 1.5	0.799
Large household members (>7 members)	1.4	0.9, 2.0	0.065
Low educational level	1.0	0.4, 2.7	0.968
No toilet at household	1.5	1.0, 2.2	0.049
Not boiling water before consumption	2.1	1.4, 3.3	0.001
Bathing in the river	1.7	1.2, 2.6	0.007
Indiscriminate defecation	1.2	0.7, 2.0	0.608
Not washing hands before eating	1.5	1.1, 2.2	0.029
Not washing hands after playing with animals	2.1	1.4, 3.1	<0.001
Not washing vegetables/fruits before consumption	1.1	0.8, 1.7	0.561
Not wearing shoes when outside	1.6	1.1, 2.4	0.012
Indiscriminate garbage disposal	1.1	0.7, 1.7	0.673
